# Dynamic photoinhibition exhibited by red coralline algae in the red sea

**DOI:** 10.1186/1471-2229-14-139

**Published:** 2014-05-20

**Authors:** Heidi L Burdett, Victoria Keddie, Nicola MacArthur, Laurin McDowall, Jennifer McLeish, Eva Spielvogel, Angela D Hatton, Nicholas A Kamenos

**Affiliations:** 1 Scottish Oceans Institute, University of St Andrews, St Andrews, UK; 2 Department of Earth and Environmental Sciences, University of St Andrews, St Andrews, UK; 3 School of Life Sciences, University of Glasgow, Glasgow, UK; 4 Scottish Association for Marine Science, Oban, Argyll, UK; 5 School of Geographical and Earth Sciences, University of Glasgow, Glasgow, UK

**Keywords:** Dimethylsulphoniopropionate (DMSP), PAM fluorometry, Maerl, Rhodolith, Coral reef, Crustose coralline algae (CCA), Photosynthesis, Photosynthetic pigment

## Abstract

**Background:**

Red coralline algae are critical components of tropical reef systems, and their success and development is, at least in part, dependent on photosynthesis. However, natural variability in the photosynthetic characteristics of red coralline algae is poorly understood. This study investigated diurnal variability in encrusting *Porolithon* sp. and free-living *Lithophyllum kotschyanum*. Measured parameters included: photosynthetic characteristics, pigment composition, thallus reflectance and intracellular concentrations of dimethylsulphoniopropionate (DMSP), an algal antioxidant that is derived from methionine, an indirect product of photosynthesis. *L. kotschyanum* thalli were characterised by a bleached topside and a pigmented underside.

**Results:**

Minimum saturation intensity and intracellular DMSP concentrations in *Porolithon* sp. were characterised by significant diurnal patterns in response to the high-light regime. A smaller diurnal pattern in minimum saturation intensity in the topside of *L. kotschyanum* was also evident. The overall reflectance of the topside of *L. kotschyanum* also exhibited a diurnal pattern, becoming increasingly reflective with increasing ambient irradiance. The underside of *L. kotschyanum*, which is shaded from ambient light exposure, exhibited a much smaller diurnal variability.

**Conclusions:**

This study highlights a number of dynamic photoinhibition strategies adopted by coralline algae, enabling them to tolerate, rather than be inhibited by, the naturally high irradiance of tropical reef systems; a factor that may become more important in the future under global change projections. In this context, this research has significant implications for tropical reef management planning and conservation monitoring, which, if natural variability is not taken into account, may become flawed. The information provided by this research may be used to inform future investigations into the contribution of coralline algae to reef accretion, ecosystem service provision and palaeoenvironmental reconstruction.

## Background

Red coralline algae (Rhodophyta: Corallinales) are found in coastal areas worldwide, encrusting rocks or growing as free-living individual thalli, which are known as maerl or rhodoliths [1]. Red coralline algae also play key roles in coastal ecosystems, providing nursery habitats for juvenile invertebrates, e.g. [2] and significantly contributing to carbonate accretion [3]. In tropical reef systems, red coralline algae act as settlement cues for coral larvae [4,5] and help to stabilise and develop tropical reef structure [3].

Interest in red coralline algae is increasing because of their potential sensitivity to projected environmental changes such as ocean acidification, e.g. [6,7], their use as a palaeoenvironmental proxy, e.g. [8-10], and their fundamental role in maintaining ecosystem function [11]. The success and development of coralline algae is, at least in part, driven by photosynthesis, yet comparatively little research has investigated their photosynthetic characteristics [12]. It is generally considered that red coralline algal photosynthesis is optimally adapted to irradiance below that typically experienced in situ [12,13], thus may be particularly susceptible to high-light induced stress [14]. Despite this, red coralline algae are found in a wide range of irradiances, from tropical coral reefs (>1500 μmol photons m^-2^ s^-1^ photosynthetically active radiation, PAR) [14,15] to the lower limit of the photic zone (>200 m, 0.0015 μmol m^-2^ s^-1^ PAR) [16]. However, under high light, thallus bleaching may occur in red coralline algae [17,18]; high light and UV radiation has also been shown to damage the DNA, photosynthetic apparatus and light harvesting pigments of non-coralline red macroalgae [19,20]. Light quality also has a significant effect on the photosynthetic capacity of red algae: blue light can stimulate pigment and protein production, whilst red light can promote growth [21].

Photosynthetic organisms often exhibit a strong diurnal cycle in photosynthetic efficiency or quantum yield (*F*_v_/*F*_m_). Such ‘dynamic photoinhibition’ reflects short-term photoacclimation mechanisms designed to minimise photo-damage during times of maximum irradiance, and to maximise photosynthesis during times of low irradiance. This is typically observed as a decrease in *F*_v_/*F*_m_ around noon, with maximum *F*_v_/*F*_m_ values in early morning and late evening, e.g. [22-24]. The extent of dynamic photoinhibition may be modified in response to the local environment, e.g. tidal exposure [25], water temperature [26] or depth [27].

Photosynthetic parameters of tropical coralline have previously been determined, e.g. [14]. However, these measurements were determined from specimens that had been maintained in a laboratory environment, which can impact the photosynthetic characteristics of red coralline algae [12]. An alternative approach is to use *in situ* fluorescence techniques, which monitor the activity of photosystem II, rather than providing a direct measurement of photosynthetic rate [28]. Pulse amplitude modulation (PAM) fluorescence provides a non-invasive method for assessing the photosynthetic characteristics of photosynthetic organisms, and has been successful applied *in situ* on red coralline algae [12,17,29].

Rapid light curves (RLCs) have become well established in the fluorescence literature and may be preferable to traditional light curves because of their short run time [30,31]. During a RLC, photosynthetic organisms are exposed to short periods of increasing levels of irradiance interspersed with short, saturating actinic pulses. RLCs thus provide fluorescence information from limiting levels of irradiance through to saturating levels, yielding a proxy for electron transport rate (ETR) through photosystem II, although the irradiance absorption of the organism and division between photosystems should be taken into account [32]. Photosynthesis-irradiance-type curves derived from RLC data permit the calculation of photosynthetic parameters including maximum (dark-adapted) and effective (light-adapted) quantum yield of fluorescence and the light saturation coefficient (the minimum saturation intensity, *E*_k_). However, unlike traditional light curves, a steady-state is not achieved during RLCs, thus results represent actual, rather than optimal, photosynthetic state, enabling relative changes in photosynthetic state across diurnal periods to be determined [30].

Dimethylsulphoniopropionate (DMSP) is a sulphur compound produced by most marine algae for numerous cellular functions [33], and is derived from methionine [33], an indirect product of photosynthesis [34]. DMSP is also the major precursor to dimethylsulphide (DMS), a biogenic gas which has been linked to local climate regulation through the formation of atmospheric aerosols and subsequent cloud development [35,36]. Red coralline algae are known to contain high concentrations of intracellular DMSP [6,37] and, given that coralline algae may often be exposed to light saturating conditions, particularly in tropical regions, the proposed role of DMSP as an antioxidant [38] may be important. The diurnal regulation of intracellular DMSP concentrations in red coralline algae is currently unknown, but recent research shows that other tropical macroalgae may up-regulate intracellular DMSP concentrations in response to night-time reductions in carbonate saturation [15].

It is important to understand the natural variation in red coralline algal photosynthetic characteristics and their potential for minimising photo-damage. Such information is particularly informative when considering the contribution made by red coralline algae in carbonate reef accretion, ecosystem service provision and palaeoenvironmental reconstructions. In that context, this study characterised the photosynthetic characteristics, pigment composition and intracellular DMSP concentrations of two tropical red coralline algae species across a diurnal period. It was hypothesised that, where algae were exposed to diurnal changes in irradiance, photosynthetic and DMSP measurements would also respond with a diurnal pattern, indicating dynamic photoinhibition and supporting the putative antioxidant function for DMSP.

## Results

### Dark-acclimation

**Figure 1:**
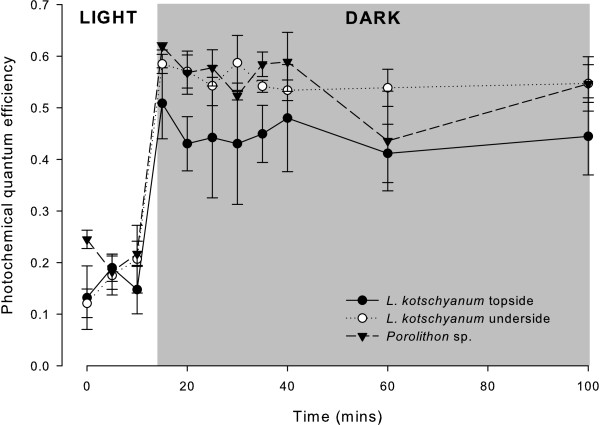
**Dark acclimation of *Lithophyllum kotschyanum *and *Porolithon *sp.** Photochemical quantum yield in the light (white background) and in the dark (grey shading) of the topside (black circles) and underside (open circles) of *L. kotschyanum* thalli and the upper surface of *Porolithon* sp. crusts (black triangles). Darkness occurred at 14:50, thus the measurement at 15 minutes represents 10 second of 'quasi' dark-acclimation. Data presented as mean ± SD.

Quantum yield was lowest in the light for *Lithophyllum kotschyanum* (topside: 0.16 ± 0.05, underside: 0.17 ± 0.06, mean ± SD) and *Porolithon* sp. (0.21 ± 0.04) (Figure 1). After 10 s of ‘quasi’ dark-acclimation, photochemical quantum yield (*F*_v_/*F*_m_) increased in both *L. kotschyanum* (topside: 0.45 ± 0.08, underside: 0.56 ± 0.03) and *Porolithon* sp. (0.57 ± 0.05). No significant difference between quantum yield measurements from t + 15 mins (‘quasi’ dark-acclimation) and t + 100 mins was observed (*L. kotschyanum* topside: p = 0.38, underside: p = 0.38, *Porolithon* sp.: p = 0.08; Figure 1).

### Photosynthetic characteristics

#### Maximum quantum yield, *F*_q_**ˈ**/*F*_m_**ˈ**_max_

**Figure 2:**
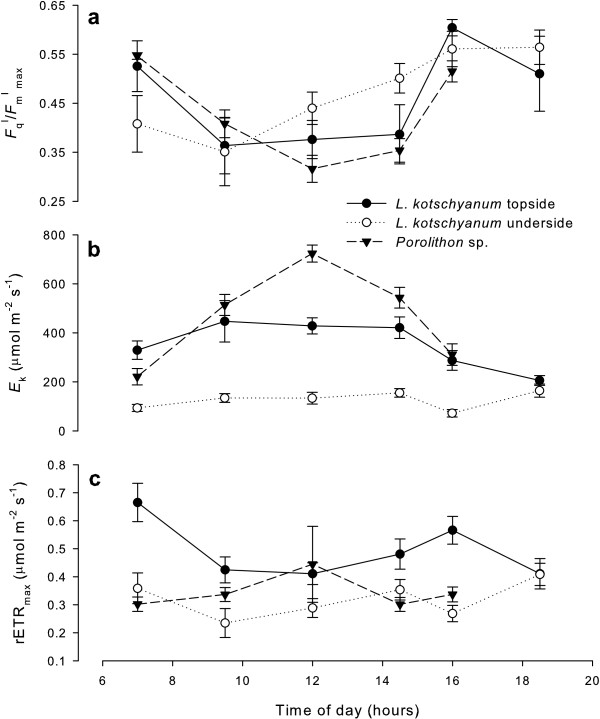
**Diurnal photosynthetic characteristics of *Lithophyllum kotschyanum *and *Porolithon *sp.** Photosynthetic characteristics of the topside (black circles) and underside (open circles) of *L. kotschyanum* thalli and the upper surface of *Porolithon* sp. (black triangles) over a diurnal cycle: **(a)** maximum photochemical quantum yield (*F*_q_ˈ/*F*_m_ˈ_max_), **(b)** minimum saturation intensity (*E*_k_, μmol photons m-2 s-1), **(c)** maximum relative electron transport rate (rETR_max_, μmol electron m-2 s-1). Data presented as mean ± SE.

In both *Porolithon* sp. and the topside and underside of *L. kotschyanum*, *F*_q_ˈ/*F*_m_ˈ_max_ was highest at dawn and dusk (~0.5) and lowest at midday (~0.3). No significant difference between the three algal morphotypes was observed at 07 h00 (F_2_ = 2.76, p = 0.103) or 12 h00 (F_2_ = 3.40, p = 0.068; Figure 2a).

#### Minimum saturation intensity, *E*_k_

At 07 h00, the *E*_k_ of *Porolithon* sp. and the topside of *L. kotschyanum* was significantly higher than the underside of *L. kotschyanum* (F_2_ = 15.21, p = 0.001; Figure 2b). At 12 h00, the *E*_k_ of *Porolithon* sp. was significantly higher than both sides of *L. kotschyanum*, and the *E*_k_ of the topside of *L. kotschyanum* was significantly higher than the underside (F_2_ = 91.28, p < 0.001; Figure 2b). The underside of *L. kotschyanum* exhibited no diurnal *E*_k_ response; *E*_k_ remained ~100 μmol photons m^-2^ s^-1^ throughout the day (Figure 2b). In contrast, the topside of *L. kotschyanum* was characterised by an increase in *E*_k_ to ~400 μmol photons m^-2^ s^-1^ by 09 h30, followed by a decline from 14 h30 to ~200 μmol photons m^-2^ s^-1^ (Figure 2b). *Porolithon* sp. was characterised by the largest diurnal pattern in *E*_k_: maximum *E*_k_ was observed at 12 h00 (~700 μmol photons m^-2^ s^-1^), followed by an afternoon decline (Figure 2b).

#### Maximum rETR, rETR_max_

No diurnal pattern in calculated rETR_max_ on the underside of *L. kotschyanum* was observed, and was maintained below the topside of *L. kotschyanum* (Figure 2c). Interestingly, contrasting diurnal patterns were observed for the topside of *L. kotschyanum* (minimum at 12 h00) and *Porolithon* sp. (maximum at 12 h00, Figure 2c). At 07 h00, rETR_max_ of the topside of *L. kotschyanum* was significantly higher than *Porolithon* sp. and the underside of *L. kotschyanum* (F_2_ = 12.52, p = 0.001). In contrast, at 12 h00, no significant difference between the algal morphotypes was observed (F_2_ = 1.23, p = 0.326).

#### Pigment composition

**Figure 3:**
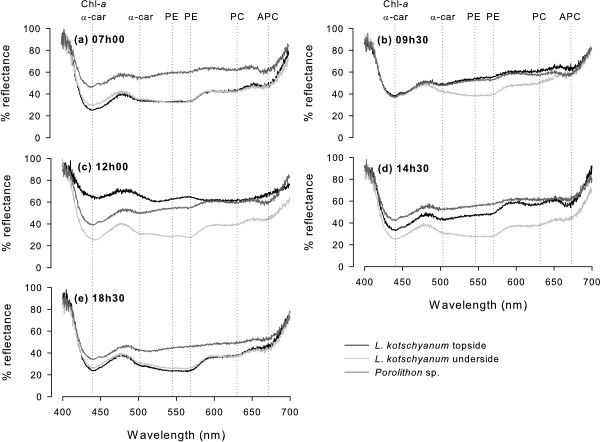
**Diurnal reflectance spectra of *Lithophyllum kotschyanum *and *Porolithon *sp.** Reflectance spectra of topside (black line) and underside (light grey line) of *L. kotschyanum* and the upper surface of *Porolithon* sp. (dark grey line) at **(a)** 07 h00, **(b)**, 09 h30, **(c)** 12 h00, **(d)** 14 h30 and **(e)** 18 h30. Dotted vertical lines indicate the absorbance peaks of photosynthetic pigments: chlorophyll-*a* (Chl-*a*), α-carotenoids (α-car), phycoerythrin (PE), phycocyanin (PC) and allophycocyanin (APC).

Peaks in absorbance (characterised by a decline in reflectance) were observed at wavelengths expected for Rhodophyta pigments according to Hedley and Mumby [39]: Chl-*a* and α-carotenoids (435–445 nm), α-carotenoids (500 nm), phycoerythrin (576 nm), phycocyanin (618 nm) and allophycocyanin (654 nm) (Figure 3). Pigment absorbance was pronounced from the underside of *L. kotschyanum* throughout the day, whilst spectra from the topside of *L. kotschyanum* spectra were flatter at 09 h30 and 12 h00 (Figure 3b,c). *Porolithon* sp. spectra exhibited the weakest absorbance, particularly at wavelengths indicative of phycoerythrin, phycocyanin and allophycocyanin (Figure 3).

The overall reflectance from *Porolithon* sp. and the underside of *L. kotschyanum* did not change throughout the day (40-60% and 20-40% respectively, Figure 3). In contrast, the overall reflectance from the topside of *L. kotschyanum* exhibited a diurnal cycle: reflectance at dawn and dusk was similar to the thallus underside; reflectance progressively increased towards 12 h00 to a maximum of 60-80% (Figure 3c).

#### Intracellular DMSP

**Figure 4:**
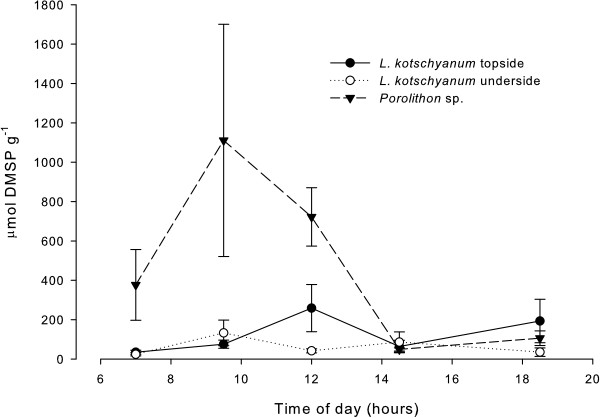
**Diurnal intracellular DMSP concentrations in *Lithophyllum kotschyanum *and *Porolithon *sp.** Intracellular DMSP concentrations (μmol g^-1^) of the topside (black circles) and underside (open circles) of *L. kotschyanum* and the upper surface of *Porolithon* sp. (black triangles) over a diurnal cycle. Data presented as mean ± SE.

The underside of *L. kotschyanum* exhibited no diurnal pattern in intracellular DMSP concentrations (Figure 4). The topside of *L. kotschyanum* was characterised by a modest increase in intracellular DMSP concentrations at 12 h00 (258 ± 120 μmol g^-1^, mean ± SE, Figure 4). Intracellular DMSP concentrations in *Porolithon* sp. were comparable to *L. kotschyanum* at 07 h00 (H_2_ = 3.84, p = 0.147), but intracellular DMSP concentrations were significantly higher in *Porolithon* sp. at 12 h00 (H_2_ = 11.63, p = 0.003, Figure 4).

## Discussion

The ability of red coralline algae to colonise the shallow photic zone in tropical regions such as the Red Sea relies on efficient photosynthetic and photoprotective mechanisms that minimise photodamage, whilst maximising photosynthetic potential, from the naturally high irradiance levels. This study highlights inter- and intra-species specific differences in *in situ* photoacclimation, pigment composition, thallus reflectance and intracellular DMSP concentrations; factors that contribute to the survival, growth and development of coralline algae in high-irradiance habitats.

### Dynamic photoinhibition

Varying degrees of dynamic photoinhibition were observed in this study. Significant diurnal patterns in photosynthetic characteristics, overall reflectance and intracellular DMSP concentrations were observed in *Porolithon* sp. and the topside of *Lithophyllum kotschyanum* thalli, suggesting that these algal morphotypes exhibited a high level of dynamic photoinhibition. Rhodophyta pigments were also less clear in the spectra of *Porolithon* sp., suggesting that the photosynthetic apparatus may be modified compared to *L. kotschyanum* to minimise photodamage. These factors may have been adopted by *Porolithon* sp. because of the alga’s position on the reef platform. The reef crest is shallow (0.5 m) and more exposed to wave action than the reef flat, which may cause localised irradiance enhancement [14]. Further, the reef crest may be periodically exposed to the air during spring tides (Burdett, pers. obs.). Such conditions necessitate efficient dynamic photoinhibition strategies that may be rapidly regulated in response to the highly variable diurnal light field, minimising photodamage, whilst optimising photosynthesis.

Efficient dynamic photoinhibition strategies will allow red coralline algae to tolerate, rather than be inhibited by, the high irradiances found in the shallow waters of the tropics, enabling the successful development of coralline algae in tropical reef systems. Previous research, which involved prolonged periods of time in the laboratory [14], may have underestimated the magnitude of dynamic photoinhibition in red coralline algae, because of coralline algal sensitivity to laboratory culture [12]. It should also be noted that a reduction in quantum yield during periods of high irradiance does not imply a reduction in net photosynthesis [40]. This, together with the presence of antioxidant compounds such as DMSP and carotenoids, may explain why coralline algae are found throughout the world’s photic zone, despite their apparent low-light adaptation [13].

### Intra-species differences

The topside and underside of *L. kotschyanum* thalli were visually different in their pigmentation, and this was evident in their overall reflectance and *E*_k_. Interestingly, the overall reflectance of the topside of *L. kotschyanum* thalli varied throughout the day, becoming most reflective during times of highest irradiance, another potential dynamic photoinhibition strategy. The shaded underside of *L. kotschyanum* was able to maintain pigmentation and did not exhibit a photosynthetic diurnal response in terms of *E*_k_, which remained low throughout the day. This suggests that the underside of *L. kotschyanum* was lower-light acclimated, in a similar manner to self-shaded branch bases in the temperate coralline alga *Lithothamnion glaciale*[12]. However, the underside of *L. kotschyanum*, whilst not exposed to full ambient PAR levels, may have received some light via seabed reflectance, and thus some modest diurnal irradiance patterns, perhaps explaining the observed diurnal patterns in *F*_q_'/*F*_m_'_max_ and rETR_max_. The carbonate sand of Suleman reef is likely to be highly reflective given its coral source; coral skeletons (even when powdered) can reflect ultraviolet radiation as yellow light, maximising photosynthesis within coral tissues [41]. Additionally, the underside of *L. kotschyanum* may periodically receive ambient PAR via thallus rolling, although, given the stark differences in pigmentation, the rate of thallus rolling is likely to be low.

### Diurnal production of antioxidant compounds

*Porolithon* sp., which exhibited the greatest photosynthetic diurnal changes, also exhibited a diurnal regulation of intracellular DMSP concentrations. The highest concentrations were observed when irradiance was highest. This is in contrast to other Red Sea macroalgae, which up-regulate intracellular DMSP concentrations in response to night-time reductions in carbonate saturation state [15]. However, both high irradiance and low saturation state can induce oxidative stress, supporting the putative antioxidant function of DMSP and its breakdown products [38]. Given the apparently high requirement for dynamic photoinhibition strategies in *Porolithon* sp., it may be supposed that any response to varying carbonate saturation is masked by the effect of large variations in day-time irradiance. Although not measured in this study, UV penetration is also high in the Red Sea [42] and may have been elevated at the reef crest, further necessitating a requirement for intracellular antioxidants.

## Conclusions

This study highlights the ability of red coralline algae to tolerate high levels of irradiance through dynamic photoinhibition strategies that may have been previously underestimated. Although high irradiance is not the only factor that may affect the success of coralline algae (e.g. grazing pressure, water temperature, carbonate chemistry), the growth and survival of coralline algae is dependent on photosynthesis. Importantly for conservation and reef management, significant diurnal variations may be observed and the colour of the algae does not necessarily reflect the algae’s photosynthetic or photoprotective capacity (*Porolithon* sp. was paler than the topside of *L. kotschyanum*). Nutrients are generally limiting in the Red Sea [43], which may mean that sulphur-containing metabolites such as DMSP are favoured over other metabolites (e.g. glycine or betaine, which contain nitrogen), allowing nitrogen to be used elsewhere in the cells, e.g. in protein synthesis [33]. Thus, in the Red Sea, DMSP may play a more important metabolic and ecological role than in other regions; this and other studies [15] suggest DMSP provides protection against irradiance- and carbonate saturation-induced oxidative stress. This has implications for the future success of coralline algae in tropical reef systems, as carbonate saturations states are projected to decline [44] and UV irradiation is projected to increase [45]. The methods used in this study, particularly the spectral reflectance and PAM fluorometry are simple to conduct, non-destructive and, in the case of PAM fluorometry, may be conducted *in situ* and thus may be suitable for tropical reef management and conservation studies. This research highlights the importance of understanding natural variability in the photosynthetic and biochemical characteristics of coralline algae when assessing potential for reef accretion, ecosystem service provision and palaeoenvironmental reconstructions by coralline algae.

## Methods

### Sampling location

Measurements were taken from the two most common red coralline algae found on the Suleman Reef, Sinai Peninsula, Egypt (28°28.79'N, 34°30.83'E): free-living *Lithophyllum kotschyanum* and encrusting *Porolithon* sp. Fluorescence measurements were taken *in situ* in November 2011 using snorkelling; other measurements were conducted on shore by hand-collecting specimens.

The fringing Suleman reef was characterised by a 100 m wide reef flat (0.5 – 1.5 m deep) dominated by macroalgae (including *L. kotschyanum*), a reef crest (0.5 m deep, primarily encrusted with *Porolithon* sp.) at the edge of the flat (~20 m wide) and a steep reef slope to 8 m depth, dominated by massive (e.g. *Porities* spp.) and branching (e.g. *Acropora* spp.) corals. Free-living *L. kotschyanum* thalli (i.e. in the form of a rhodolith) were characterised by bleached topsides and pigmented, dark pink undersides (Additional file 1: Figure S1). *Porolithon* sp. crusts were uniformly light pink.

### *In situ* irradiance

*In situ* PAR (μmol photons m^-2^ s^-1^) was measured using an Apogee QSO-E underwater quantum sensor and a Gemini voltage data logger over a full diel cycle. PAR is not significantly different between the reef flat and reef crest on Suleman Reef [15]. Maximum PAR was between 10 h00 and 12 h00 (~800 – 900 μmol m^-2^ s^-1^).

### Pigment composition

The reflectance spectra of the topside and underside of *L. kotschyanum* and the upper surface of *Porolithon* sp. were used to identify the pigment composition of the algal cells (all samples were from independent thalli for topside, underside and encrusting measurements). Coralline algal samples (n = 3 – 7 due to sample availability) were collected from the reef and stored at ambient conditions for no more than 20 minutes before analysis. Coralline algal samples were patted dry and immediately exposed to directed light (Scubapro Nova Light 230 torch, spectral range: 380–750 nm) via a 5 mm fibre optic cable (Walz GmbH, Effeltrich, Germany). Reflected light was transmitted to a USB 2000+ Ocean Optics spectrometer (Dunedin, USA) via a 400 μm fibre optic cable (Ocean Optics) and the reflectance spectra recorded. Due to the uneven surface of the samples, it was logistically difficult to maintain a fixed angle between the two fibre optic cables. Instead, for each sample the cables were positioned to achieve maximum reflectance based on the real-time spectrometer trace. Percentage absorbance was calculated based on the difference between sample absorbance and that from a white standard (100% reflectance, spectra recorded every 5 samples). The absorbance wavelengths of Rhodophyta pigments were obtained from Hedley and Mumby [39].

### Fluorescence measurements

Chlorophyll-*a* fluorescence measurements were, where possible, conducted *in situ* using a Diving-PAM fluorometer (Walz GmbH, Effeltrich, Germany). Measurements were taken using the methodology described by Burdett et al. [12], using a 5 mm diameter fibre optic cable. The fluorescence notation used throughout this manuscript follows that of Burdett et al. [12]; a notation table is provided as supplementary information (Additional file 2: Table S1). In a fully relaxed, dark-acclimated state, the minimum and maximum fluorescence yields are termed *F*_o_ and *F*_m_ respectively. These parameters are termed *F*_o_' and *F*_m_' respectively under actinic light.

### Dark-acclimation

The suitability of a short dark acclimation period was assessed for both the topside and underside of *L. kotschyanum*, and the upper surface of *Porolithon* sp. Samples (n = 3) were collected from Suleman reef and maintained in the laboratory at ambient conditions (all samples were from independent thalli). Under ambient light, the effective quantum yield (*F*_q_'/*F*_m_') of the thalli was determined by exposing the thalli to 3 saturating light pulses at 5 min intervals (t = 0, +5 and +10 min). After 14 min 50 s, the thalli were placed in darkness and 8 further saturation pulses were conducted at t + 15, 20, 25, 30, 35, 40, 60 and 100 mins, representing maximum quantum yield (*F*_v_/*F*_m_). Thus, at the 15 minute measurement, the algae had been exposed to 10 seconds of darkness, so called 'quasi' dark-acclimation [30]. Saturation pulses were taken from the same thallus location at each timepoint. As has been observed in temperate red coralline algae [12], *F*_v_/*F*_m_ derived from 10 s of ‘quasi’ dark-acclimation (t + 15 mins measurement) was not significantly different to *F*_v_/*F*_m_ at t + 100 mins (full dark-acclimation – time in darkness: 85 mins, 10 seconds; Mann–Whitney comparisons: *L. kotschyanum* topside: p = 0.38, *L. kotschyanum* underside: p = 0.38, *Porolithon* sp.: p = 0.08, Figure 1), suggesting that ‘quasi’ dark acclimation was sufficient for obtaining *F*_o_ and *F*_m_ fluorescence measurements.

### Rapid light curves

RLCs (n = 5) were conducted on the topside and underside of *L. kotschyanum*, and on *Porolithon* sp. at six times throughout the diurnal cycle: 07 h00 (ambient PAR: 174 μmol photons m^-2^ s^-1^), 09 h30 (755 μmol photons m^-2^ s^-1^), 12 h00 (814 μmol photons m^-2^ s^-1^), 14 h30 (421 μmol photons m^-2^ s^-1^), 16 h00 (82 μmol photons m^-2^ s^-1^) and 18 h30 (dark) (all samples were from independent thalli). All RLCs were conducted after 10s of ‘quasi’-dark acclimation as this had previously been determined to be sufficient time to achieve maximum yield measurements (Figure 1). Actinic light illumination was increased over nine incremental PAR intensities; *L. kotschyanum*: 0, 135, 230, 346, 493, 731, 997, 1455, 2125 μmol photons m^-2^ s^-1^; Porolithon sp.: 0, 387, 548, 825, 1126, 1719, 2504, 3710, 6061 μmol photons m^-2^ s^-1^. Logistical constraints prevented RLCs from being conducted *in situ* at 18 h30. Instead, *L. kotschyanum* thalli were collected by hand using snorkelling and stored in the dark at ambient conditions for no more than 20 minutes before the RLCs were run. *Porolithon* sp. RLCs were not be conducted at 18 h30.

Each RLC produced a series of quantum yield measurements that were fitted against the following model to describe the light response of quantum efficiency using non-linear least squares regression [12,46]:

(1) $$F_{q}ˈ/F_{m}ˈ=\left[\left(F_{q}ˈ/F_{m}ˈ\times E_{k}\right)\left(1–\exp\left(–E/E_{k}\right)\right)\right]/E$$

where *E*_k_ is the minimum saturation intensity (μmol photons m^-2^ s^-1^) [47] – the light intensity where light shifts from being photosynthetically limiting to photosynthetically saturating. *E* is equivalent to the RLC PAR (μmol photons m^-2^ s^-1^). For the first step of the RLC, where the algae were quasi dark-acclimated, *F*_v_/*F*_m_ was used instead of *F*_q_'/*F*_m_'. Eqn 1 was also used to calculate the theoretical maximum quantum yield, *F*_q_'/*F*_m_'_max_. As *F*_q_'/*F*_m_'_max_ was derived from the RLC illumination, differences observed represent differences in light acclimation rather than environmental light availability [48].

Relative electron transport rate (rETR, μmol electrons m^-2^ s^-1^) was calculated from *F*_q_'/*F*_m_' measurements at each actinic light intensity (*E*) of the RLC:

(2) $$\mathrm{rETR}=F_{q}ˈ/F_{m}ˈ\times \mathrm{PAR}$$

where PAR is the RLC irradiance (μmol photons m^-2^ s^-1^). Maximum rETR (rETR_max_, μmol electrons m^-2^ s^-1^) was calculated by fitting the light-response of rETR to the following least-squares regression [46], modified from Jassby and Platt (1976) [49]:

(3) $$\mathrm{rETR}={\mathrm{rETR}}_{\text{max}}*\left[1-\exp\left(-\alpha *E/{\mathrm{rETR}}_{\text{max}}\right)\right]$$

where α is the photosynthetic rate in the light-limited part of the RLC [30]

### Intracellular DMSP

Samples (n = 5) of the topside and underside of *L. kotschyanum* and from *Porolithon* sp. crusts were collected from Suleman reef at 07 h00, 09 h30, 12 h00, 14 h30, 16 h00 and 18 h30 and immediately fixed for intracellular DMSP using 10 M sodium hydroxide in gas-tight glass vials (Wheaton) sealed with Pharma-Fix septa (Grace Alltech) (all samples were from independent thalli). All samples were stored in the dark prior to analysis of the vial headspace using a Shimadzu 2014 gas chromatograph fitted with a 25 m capillary column (Restek RTx-5MS 30 m column, 0.25 mm ID) and a sulphur-specific FPD detector (injector port and column oven temperature: 45°C, detector: 200°C). Sample concentrations were quantified from DMSP standard calibration curves (DMSP standard from Research Plus Inc.). The limit of detection was 30 nmol per injection; standard and sample precision was within 3%.

### Statistical analyses

A Mann–Whitney test was used to compare quantum yields of the three algal morphotypes at t + 15 and t + 100 mins in the dark-acclimation experiment. Differences in *F*_q_'/*F*_m_'_max_, *E*_k_ and rETR_max_ between the three algal morphotypes at 07 h00 and 12 h00 were identified using an ANOVA general linear model (test assumptions for normality [Anderson-Darling test] and homogeneity of variance [Bartlett's test] were met without data transformation; all samples were from independent thalli). Intracellular DMSP concentrations between the different algal morphotypes at 12 h00 and 18 h30 were identified using Kruskall-Wallis tests (assumptions for parametric testing could not be met). All analyses were conducted in Minitab V14.

## Competing interests

The authors declare that they have no competing interests.

## Authors’ contributions

HB, NK and AH designed the study. VK, NM, LM, JM, ES and NK collected the data. HB analysed and interpreted the data. HB wrote the manuscript; all authors contributed to the final submission. All authors read and approved the final manuscript.

## Supplementary Material

Additional file 1: Figure S1 Example of a free-living coralline algal thallus (Lithophyllum kotschyanum) from Suleman reef, Egypt with a (a) bleached topside and (b) pigmented underside. Scale bar = 5 cm. Click here for file

Additional file 2: Table S1 Fluorescence notation used within Burdett et al. Fluorescence yield have instrument-specific units, ratios are dimensionless. Click here for file
