# Supplementary material for: Dynamic photoinhibition exhibited by red coralline algae in the red sea
Source: BMC Plant Biol. 2014 May 20;14:139. doi: 10.1186/1471-2229-14-139 (PMC4032452; doi:10.1186/1471-2229-14-139)

Figure S1: Example of a free-living coralline algal thallus (*Lithophyllum kotschyanum*) from Suleman reef, Egypt with a (a) bleached topside and (b) pigmented underside. Scale bar = 5 cm.


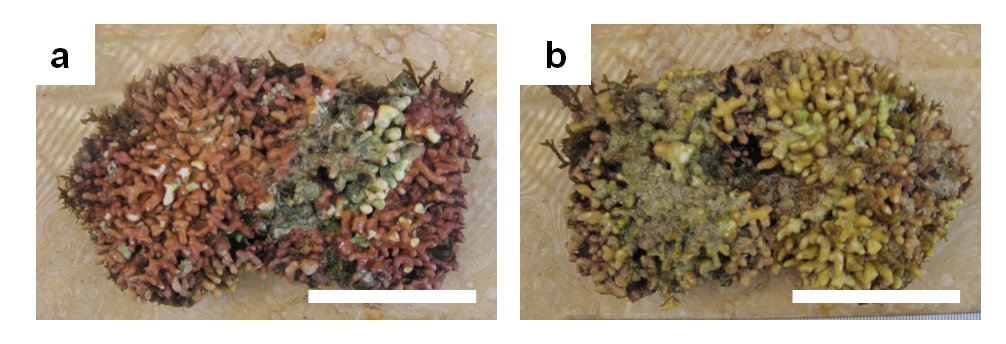

Supplement: Additional file 1: Figure S1 — Example of a free-living coralline algal thallus (Lithophyllum kotschyanum) from Suleman reef, Egypt with a (a) bleached topside and (b) pigmented underside. Scale bar = 5 cm. [file 1471-2229-14-139-S1.docx]
