# Supplementary material for: Dynamic photoinhibition exhibited by red coralline algae in the red sea
Source: BMC Plant Biol. 2014 May 20;14:139. doi: 10.1186/1471-2229-14-139 (PMC4032452; doi:10.1186/1471-2229-14-139)
Supplement: Additional file 2: Table S1 — Fluorescence notation used within Burdett et al. Fluorescence yield have instrument-specific units, ratios are dimensionless. [file 1471-2229-14-139-S2.docx]

Additional file 1: Table S1 Fluorescence notation used within Burdett et al. Fluorescence yield have instrument-specific units, ratios are dimensionless.

| **Parameter** | **Definition** |
| --- | --- |
| *F*_o_ | Minimum fluorescence (dark acclimated) |
| *F*_o_' | Minimum fluorescence (light acclimated) |
| *F*_m_ | Maximum fluorescence (dark acclimated) |
| *F*' | Fluorescence under actinic light |
| *F*_v_ | Variable fluorescence (dark acclimated); (*F*_m_ – *F*_o_) |
| *F*_v_' | Variable fluorescence yield under actinic light; (*F*_m_' – *F*_o_') |
| *F*_q_' | Fluorescence quenched; (*F*_m_ – *F*') |
| *F*_v_/*F*_m_ | Maximum quantum yield of PSII (dark acclimated) |
| *F*_q_'/*F*_m_' | Effective quantum yield of PSII under actinic light |
| *F*_q_'/*F*_m_'_max_ | Calculated maximum quantum yield |
| α | Photosynthetic rate in the light-limited part of the RLC |
| *r*ETR_max_ | Maximum relative electron transport rate (µmol electrons m^-2^ s^-1^) |
| *E_k_* | Light saturation coefficient (µmol photons m^-2^ s^-1^) |
